# Individual differences in response speed and accuracy are associated to specific brain activities of two interacting systems

**DOI:** 10.3389/fnbeh.2014.00251

**Published:** 2014-07-22

**Authors:** Rinaldo Livio Perri, Marika Berchicci, Donatella Spinelli, Francesco Di Russo

**Affiliations:** ^1^Department of Human Movement, Social and Health Sciences, University of Rome “Foro Italico”Rome, Italy; ^2^Department of Psychology, University of Rome “La Sapienza”Rome, Italy; ^3^Neuropsychological Unit, IRCCS Santa Lucia FoundationRome, Italy

**Keywords:** EEG, event related potentials (ERPs), decision making, speed-accuracy tradeoff, movement-related cortical potentials (MRCPs), Bereitschaftspotential (BP)

## Abstract

The study investigates the neurocognitive stages involved in the speed-accuracy trade-off (SAT). Contrary to previous approach, we did not manipulate speed and accuracy instructions: participants were required to be fast and accurate in a go/no-go task, and we selected *post-hoc* the groups based on the subjects’ spontaneous behavioral tendency. Based on the reaction times, we selected the fast and slow groups (Speed-groups), and based on the percentage of false alarms, we selected the accurate and inaccurate groups (Accuracy-groups). The two Speed-groups were accuracy-matched, and the two Accuracy-groups were speed-matched. High density electroencephalographic (EEG) and stimulus-locked analyses allowed us to observe group differences both before and after the stimulus onset. Long before the stimulus appearance, the two Speed-groups showed different amplitude of the Bereitschaftspotential (BP), reflecting the activity of the supplementary motor area (SMA); by contrast, the two Accuracy-groups showed different amplitude of the prefrontal negativity (pN), reflecting the activity of the right prefrontal cortex (rPFC). In addition, the post-stimulus event-related potential (ERP) components showed differences between groups: the P1 component was larger in accurate than inaccurate group; the N1 and N2 components were larger in the fast than slow group; the P3 component started earlier and was larger in the fast than slow group. The go minus no-go subtractive wave enhancing go-related processing revealed a differential prefrontal positivity (dpP) that peaked at about 330 ms; the latency and the amplitude of this peak were associated with the speed of the decision process and the efficiency of the stimulus-response mapping, respectively. Overall, data are consistent with the view that speed and accuracy are processed by two interacting but separate neurocognitive systems, with different features in both the anticipation and the response execution phases.

## Introduction

In a typical go/no-go task subjects are required to quickly respond to go trials (e.g., pressing a button) and to refrain the response to no-go trials. This task has been widely investigated because it involves many cognitive processes, such as motor preparation (Rinkenauer et al., 2004; Berchicci et al., 2012), sensory evidence accumulation (Burle et al., 2004; Perea et al., 2010), decision-making (Schall, 2001; Heekeren et al., 2008), proactive and reactive inhibition (Aron et al., 2004; Aron, 2011) and motor response. The neural basis of these processing have been investigated at various levels, from animal (Mishkin, 1964) to humans (Konishi et al., 1998; Garavan et al., 1999; Konishi et al., 1999). However, neurocognitive processes underlying the perceptual decision-making are not entirely defined, and particularly the processes supporting the trade-off between speed and accuracy of the response in the go/no-go task has received little attention.

In the context of a perceptual discriminative task, decisions can be viewed as a result of continuous accumulation of sensory information from a baseline point until reaching a threshold (Ratcliff, 1978). Fast decisions are more error prone, while careful ones take longer (Wenzlaff et al., 2011); this phenomenon is known as the speed-accuracy tradeoff (hereafter, SAT) (for a review see Bogacz et al., 2010).

The cognitive models of decision making consider the SAT as the outcome of an evidence accumulation process. One of the most important accumulation models is the Ratcliff’s diffusion model (Ratcliff and Rouder, 2000; Ratcliff, 2002; Ratcliff and Tuerlinckx, 2002; Ratcliff et al., 2004); this model considers the response execution as a result of different processes, such as the quality of evidence accumulation, the decision criteria and the stimulus encoding. This model assumes that decisions are taken through a noisy process that accumulates information over time (Ratcliff and McKoon, 2008). Differently to the Ratcliff’s model, the leaky competing accumulator model (Usher and McClelland, 2001) also considers the effects of leakage and amplification of differences (partly attributable to the noise), while the linear ballistic accumulation model (Brown and Heathcote, 2005, 2008) includes the between-trial variability in input strength and in the starting point of accumulation. Summarizing, all the accumulation models share the assumption that SAT can be explained by changes in the distance between a baseline and a threshold, so that a larger distance yield slower but more accurate responses (Reddi and Carpenter, 2000; Usher and McClelland, 2001; Bogacz et al., 2006; Simen et al., 2006). Computationally, a baseline increase would be equivalent to a threshold decrease. Despite the large amount of evidence supporting the modeling of behavioral results according to mathematical models of decision-making, the neural mechanisms for adjusting the baseline-to-threshold distance are only partially understood (Kim and Lee, 2011).

Several functional magnetic resonance imaging (fMRI) studies attempted to identify the brain regions involved in SAT by means of instructions emphasizing either response speed or accuracy; these studies used a Simon task with right/left hand response (Forstmann et al., 2008a; van Veen et al., 2008) or a cued motion direction discrimination task (Forstmann et al., 2008b, 2010; Ivanoff et al., 2008). Forstmann et al. (2008b) showed that the preparation for fast actions was associated with larger activity of the anterior striatum and the rostral part of the supplementary motor area (pre-SMA). Two other studies (Ivanoff et al., 2008; van Veen et al., 2008) confirmed that speed emphasis leads to greater activation in the striatum and pre-SMA, but showed also the involvement of other areas: the premotor area (PMA), the dorsolateral prefrontal cortex (DLPFC) and left parietal cortices. Forstmann et al. (2008a) noted individual differences in the task, i.e., under speed constraint some participants adjusted their response thresholds more than others; the participants who had a relatively large decrease in response caution also had a relatively large increase in activation for the right anterior striatum and right pre-SMA. On the other hand, none of these studies found SAT-related changes in sensory cortical areas.

This latter result was also reported by the electroencephalographic (EEG) studies on the SAT, which used tasks such as Simon, flankers and letter recognition and focused on the motor stages evaluating the lateralized readiness potential (LRP). Sangals et al. (2002) found that time pressure increased the LRP amplitude. Other studies (Osman et al., 2000; van der Lubbe et al., 2001; Rinkenauer et al., 2004) considered the LRP latency and found that the faster the response time (RT), the earlier the LRP peak. Only Brunia (2003) used a go/no-go task: they found that under speed instructions the preparatory activity was enhanced with respect to the instruction of being as fast and accurate as possible. Considering the locus of SAT, these studies concluded that SAT mechanism operated at the late motor stage, although some effects were also detected at the premotor stage (Rinkenauer et al., 2004). Finally, a recent MEG study using a face/house categorization task described the timing of the decision processing affected by SAT, and its dependence on sensory evidence (Wenzlaff et al., 2011). Emphasis on speed resulted in a higher activation of SMA and precuneus, whereas the left DLPFC showed larger activity under accuracy than speed instructions, possibly reflecting a higher level of accumulated evidence; however, they did not find SAT effects in sensory areas.

Overall, these studies provide convergent, but also divergent evidence. It is likely that the differences between results are caused by differences in tasks (such as perceptual categorization vs. Simon task), experimental designs (such as single trial cueing vs. cuing blocks), signal analyses (stimulus-locked vs. response-locked activity) and computational reference modeling. These contrasting results call for further investigations of SAT, particularly using other tasks and other analyses. To this aim, in the present study we investigated both pre- and post-stimulus SAT-related processes by means of high-density EEG and stimulus-locked analyses in a go/no-go task.

A key methodological difference with previous studies is that we did not force subjects to emphasize speed or accuracy, rather we sought to separately describe the neural processes subserving speed or accuracy on the basis of the subjects’ spontaneous behavioral tendency; thus, subjects were assigned *a posteriori* to each group (high or low accuracy; fast or slow speed) based on the observed performance. Spontaneous (idiosyncratic) employment of speed and accuracy strategies reflects, at least in part, a trait disposition (Ashcraft and Faust, 1994; Ashcraft and Kirk, 2001; Szymura and Wodniecka, 2003; Flehmig et al., 2010); thus, we thought that speed or accuracy behaviors could be better unfolded in their habitual trend. A limit of the approaches based on instructions manipulation is the individual differences in copying with the instructions themselves; for example, a spontaneously fast subject can easily behave more slowly, while a slow subject may have trouble to speed up. Consistently, these two subjects may engage different cognitive resources to fulfill with the instructions because of their basic dispositions, and this could influence the individuation of the SAT-neural correlates. Following this idea, we hypothesize that the idiosyncratic, behaviorally measured, individual speed-disposition or accuracy-disposition may reflect the dominance of a motor-related (in case of speed-oriented subjects) vs. decision making-related (in case of accuracy-oriented subjects) cortical mechanisms more clearly than it can be observed in studies manipulating either the speed or accuracy emphasis in the same subject. Moreover, we wonder whether, using this approach, speed- and accuracy-related neural processes were identifiable also at the perceptual level; this expectation was not supported by fMRI and MEG literature, because SAT-related effects were not found in sensory areas (Forstmann et al., 2008b; Ivanoff et al., 2008; van Veen et al., 2008; Wenzlaff et al., 2011). However, we hypothesize that the idiosyncratic behavioral performance can also express at perceptual processing level; this view was supported by previous event-related potentials (ERPs) evidence from our group (Di Russo et al., 2006) showing that the amplitude of the visual N1 evoked in a go/no-go task was increased in subjects with very fast RTs. Further, we expected to find a difference in the occurrence of a pre-movement brain component, which might partly explain the performance in the speed domain. Particularly, we hypothesized that this component could be represented by the prefrontal positivity (pP), previously associated to the stimulus-response (S-R) mapping process (see, e.g., Berchicci et al., 2014): indeed, if that component triggers the response execution in the go trials, a latency modulation at that level should predict the speed of the response execution, while an amplitude modulation could reflect the quality of the subserved processing, as probably reflected by the accuracy comparison.

## Methods

### Subjects

From a database of 130 subjects who participated in the go/no-go task (described below), we firstly ordered them based on the values of two behavioral parameters: (1) speed: the individual median RTs of correct trials; and (2) accuracy: the individual mean percentage of false alarms (FAs) (i.e., responses to no-go stimuli). We calculated the quartiles from each data set (i.e., mean value for the lower and upper quartiles for RTs was 524 and 372 ms respectively; mean value for the lower and upper quartiles for FAs was 18.00 and 2.18% respectively). Then, we selected the groups of subjects falling into the lower (33 and 32 subjects for speed and accuracy, respectively) and upper (32 and 34 subjects for speed and accuracy, respectively) quartiles. Afterwards, the groups were matched for age, gender and, most important, for the value of the other reference parameter, i.e., the two Speed-groups were FA-matched, and the two Accuracy-groups were RT-matched. Finally, we selected 63 participants for the final groups, each of one was composed by about 21 subjects (see Table 1); 23 of them belonged to two groups. Obviously, the main risk of this approach is that it does not allow a perfect groups match according to demographic and behavioral data, but it is important to note that in the final groups the statistical differences were significant between the reference parameter only (see Table 1). The demographic and behavioral data of the four groups and their relative comparisons (performed by *t*-test) are also shown in Table 1. The participants had normal or corrected-to-normal vision and no history of neurological or psychiatric disorders; all of the subjects were right-handed (Edinburgh handedness inventory; Oldfield, 1971). After explanations of the procedures, all of the participants provided written informed consent, approved by the local ethical committee.

**Table 1:** **Comparison of demographic and behavioral data in the Speed- and Accuracy-groups**.

	**Speed**			**Accuracy**		
	**Fast**	**Slow**	***t* (*p*-value)**	**Accurate**	**Inaccurate**	***t* (*p*-value)**
***N*. (males)**	23 (18)	22 (15)		20 (14)	21 (16)	
**Age (SD)**	34.4 (10.3)	39.9 (11.3)	−1.7 (>0.05)	34.3 (12.2)	33.6 (13.4)	0.17 (>0.05)
**RT (SD)**	388 (34)	489 (30)	**−10.7 (<0.0001)**	435 (47)	413 (57)	1.4 (>0.05)
**FA (SD)**	10.3 (7.7)	7.1 (5.3)	1.6 (>0.05)	2.3 (1.2)	15.4 (5.9)	**−9.7 (<0.0001)**

*Age is expressed years, RT in milliseconds and FA in percentage*.

### Procedure and task

Subjects were tested in a sound attenuated, dimly lit room; they were comfortably seated in front of a computer screen at a distance of 114 cm, and a board was fixed on the armchair allowing them to push freely the button panel positioned on it. Four visual stimuli (i.e., four squared configurations made by vertical and horizontal bars) were randomly presented for 260 ms with equal probability (*p* = 0.25). Two stimuli were defined as targets (go stimuli, *p* = 0.5), the other two were defined as non-targets (no-go stimuli, *p* = 0.5). The stimulus-onset asynchrony varied from 1 to 2 s to avoid time prediction effects on the RTs (for more details on the paradigms, see Berchicci et al., 2012). All of the participants were asked to be very accurate in discriminating the stimuli and to press the button as fast as possible with the right hand when a target appeared on the screen (go stimuli) and withhold the response when a non-target appeared (no-go stimuli). A minimum of 400 trials were recorded for both go and no-go stimuli.

### Electrophysiological recording and data analysis

The EEG signal was recorded using BrainVision^TM^ system (Brain-Products GmbH, Munich, Germany) with 64 electrodes mounted according to the 10–10 international system. All electrodes were referenced to the left mastoid. Horizontal and vertical electrooculogram (EOG) were also recorded using electrode at the right external canthi and below the left eye, respectively. Electrode impedances were kept below 5 KΩ. The EEG was digitized at 250 Hz, amplified (band-pass of 0.01–80 Hz including a 50 Hz notch filter) and stored for offline averaging. Artifact rejection was performed prior to signal averaging to discard epochs contaminated by blinks, eye movements or other signals exceeding the amplitude threshold of ±100 μV.

In order to investigate both the pre- and the post-stimulus activities, the artifact-free signals were separately segmented into go and no-go trials, and then averaged in 2000 ms epochs (from 1100 ms before to 900 ms after the stimulus onset). The baseline was defined as the mean voltage during the initial 200 ms of the averaged epochs. To further reduce high frequency noise, the averaged signals were low pass filtered (i.e., Butterworth) at 25 Hz (slope 24 dB/octave). All of the statistical analyses were separately performed for Speed- and Accuracy-groups.

### Pre-stimulus activities

Statistical differences in the pre-stimulus mean amplitude of Speed- and Accuracy-groups were initially assessed with sample-by-sample *t*-test in all electrodes in order to select the locations and the time windows where the differences were consistently significant.

For the Speed-groups, the mean amplitude on Cz derivation in the −500/0 time window, reflecting the Bereitschaftspotential (BP), was submitted to a repeated measures ANOVA with Group (Fast vs. Slow) and Condition (go vs. no-go) as factors.

Based on preliminary analysis on the Accuracy-groups, we selected the following electrodes on the left (AF3-F3-F7-FC5) and right (AF4-F4-F8-FC6) prefrontal cortex (PFC); the ERPs recorded at these electrodes were averaged in order to obtain a representative pool of activities in each hemisphere of the PFC. The mean amplitude between 250 ms before and 50 ms after stimulus onset at the two selected pools was submitted to a 2 × 2 × 2 ANOVA(Group × Pool × Condition). *Post-hoc* comparisons were conducted using Fisher’s least significant difference (LSD) test. Furthermore, the correlation coefficients (Pearson’s *r* coefficients) were performed between behavioral and pre-stimulus electrophysiological data for the Speed- and Accuracy-groups. The overall *α*-level was fixed at 0.05.

### Post-stimulus activities

Based on the peak electrodes, the typical post-stimulus ERPs components were measured as follows: the P1 on PO8, the N1 on PO7, the N2 on Cz, and the P3 on Pz and Cz in the go and no-go condition, respectively. The peak amplitude and latency of these components were submitted to separate 2 × 2 ANOVAs with Group (Fast vs. Slow or Accurate vs. Inaccurate) as between factor and Condition (go vs. no-go) as within factor. *Post-hoc* comparisons were conducted using Fisher’s LSD test. The correlation coefficients (Pearson’s *r* coefficients) were performed between behavioral and post-stimulus electrophysiological data; further, in order to look for the relationship between pre- and post-stimulus neural activities in the decision-making process, we also performed the correlation analyses between the electrophysiological data in both Speed- and Accuracy-groups. The results of analyses will be reported only when they are significant (*p* < 0.05).

### Differential waves

In a study combining EEG and fMRI measures (Di Russo et al., 2013b), it was showed that stimulus perception in the go/no-go task triggers early activity in anterior insula, corresponding to the pP component of the EEG. The positivity enhancement over the frontopolar derivations was closely associated to the go condition as triggering the response execution (Berchicci et al., 2014): it started bilaterally 80 ms after the stimulus and peaked at 300–350 ms, as also reported in a study with neurological patients (Di Russo et al., 2013a).

In the present study, to better isolate the pP component, we adopted the differential method subtracting the individual no-go ERP from the go ERP of the same subject; the individual subtraction waves were then separately averaged for Speed- and Accuracy-groups. Obviously, the risk in adopting this method is to indistinctly subtract different activities taking place in the same period. In order to avoid this, we limited our analyses on the Fp1 and Fp2 sites in the time window following the stimulus appearance. This method was motivated by the fact that we wanted to emphasize the prefrontal positive activity, expecting to find latency modulations as a consequence of difference in response speed. We also looked at that component in the accuracy-groups, in which the speed-match should not produce a modulation in the peak latency.

The data were band pass filtered (1–20 Hz; slope 24 dB/octave) to reduce the low-frequency noise and to facilitate the peak detection. The visual inspection of the averaged differential waves showed a positive peak at approximately 330 ms bilaterally over the frontopolar electrodes (i.e., Fp1 and Fp2); since both topography and latency of this difference wave were similar to that of the pP elsewhere reported (Di Russo et al., 2013a,b; Berchicci et al., 2014), this component will be called differential prefrontal positivity (dpP) wave.

The onset latency (calculated as the first deflection larger than twice the absolute value of the baseline mean) and the peak amplitude and latency of the dpP were submitted to 2 × 2 ANOVAs with Group and Site (Fp1, Fp2) as factors, repeated for both Speed- and Accuracy-groups. The correlation coefficients (Pearson’s *r* coefficients) were computed between behavioral and dpP data. The overall *α*-level was fixed at 0.05.

## Results

**Figure 1:**
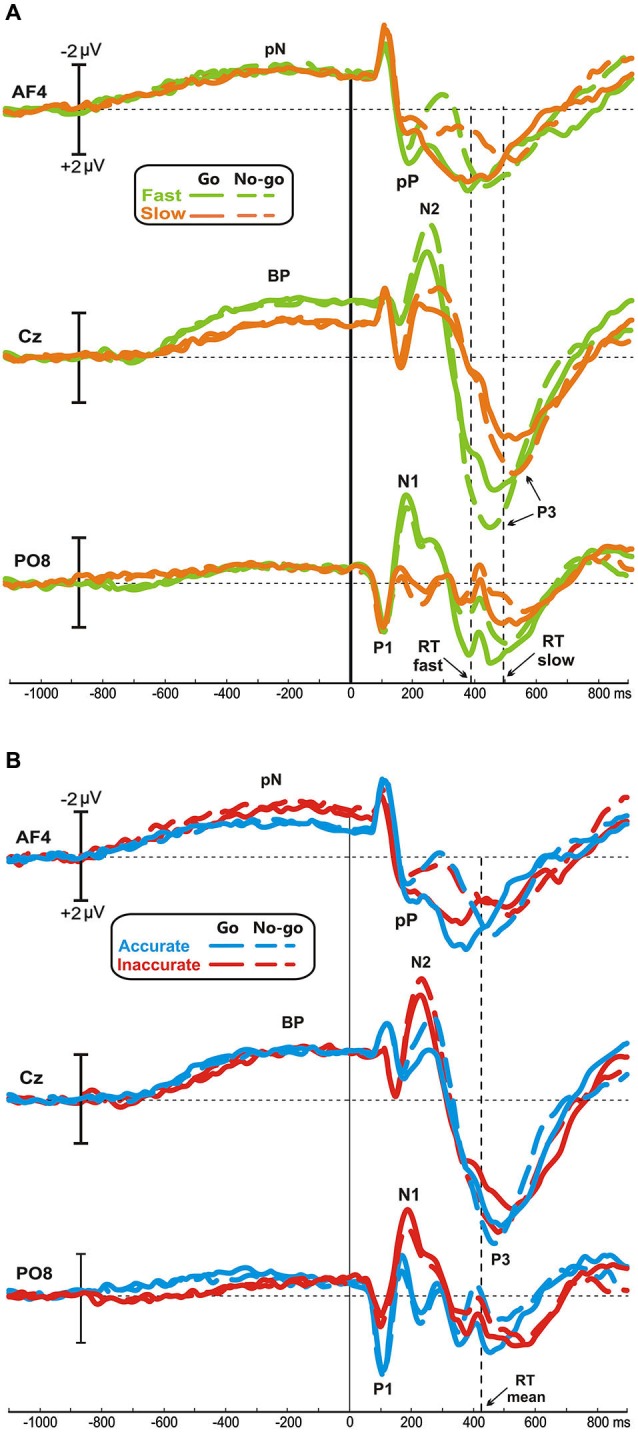
**Grand averaged waveforms of Speed- (A) and Accuracy-groups (B) in the three relevant sites (AF4, Cz, PO8); time 0 corresponds to the stimulus onset**. The different groups and task conditions are superimposed with different colors. pN, prefrontal negativity; BP, Bereitschaftspotential.

Figure 1 illustrates the ERP waveforms of both Speed- (Figure 1A) and Accuracy-groups (Figure 1B) at three relevant sites (AF4, Cz, PO8) for both go and no-go conditions. Time 0 represents the stimulus onset; inspection of the figure indicates that these stimulus-locked ERPs using long pre-stimulus analysis allow to appreciate the motor preparation activity, which is usually obtained by the motor response-locked ERPs, called movement-related cortical potentials (MRCPs).

### Pre-stimulus activities

**Figure 2:**
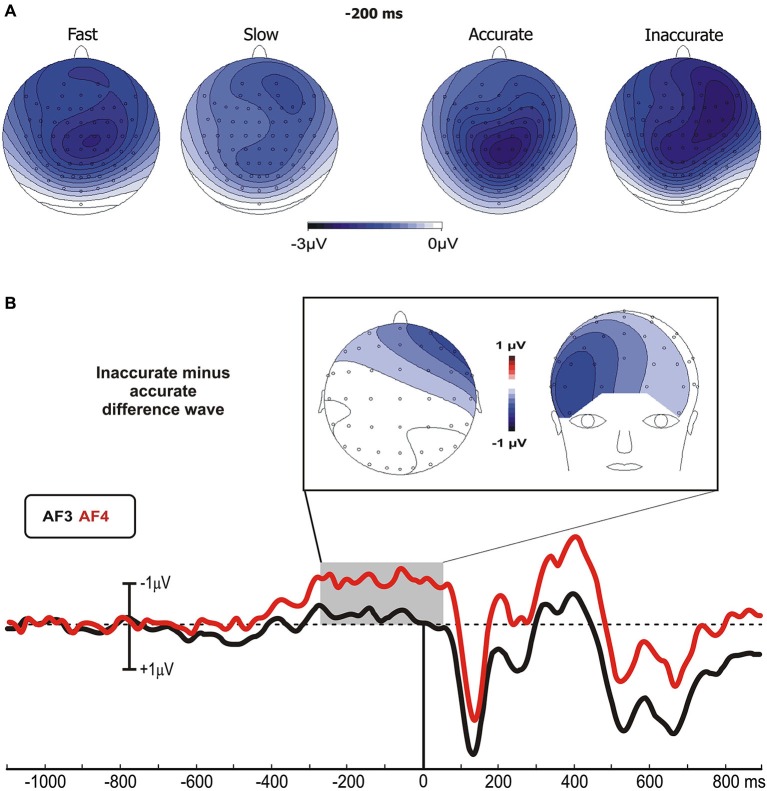
**(A)** Scalp topographies (top-flat view) of the grand averaged pre-stimulus activities in the Speed- and Accuracy-groups. **(B)** Topographical distribution in the −250/+50 ms time window and waveforms at relevant sites of the left and right PFC pools of the differential activity in the Accuracy-groups (inaccurate minus accurate group). Time 0 corresponds to the stimulus onset.

No differences were found between go and no-go conditions before stimulus onset. In all groups, the prefrontal negativity (pN) started about 800 ms before the stimulus appearance (see AF4); 200 ms later, over Cz, emerged the BP that progressively raised reaching its maximum at about 300 ms before the stimulus onset. The BP component was larger in the fast than the slow group, while the two Accuracy-groups had identical BP component. By contrast, the pN was modulated by the accuracy only, i.e., the inaccurate group showed a larger negativity than accurate group. Figure 2A shows the topographical distribution of the aforementioned pre-stimulus activities. The activity over the medial frontal-central areas (likely the SMA) in the fast group was larger than the slow group; on the other hand, the inaccurate group showed a greater negativity than the accurate over the PFC, especially in the right-hemisphere. In order to visually enhance the presence of hemispheric differences in the inaccurate group, Figure 2B shows the differential waves obtained over lateral PFC by subtraction of the grand averaged ERP of accurate group from that of the inaccurate.

With regards to the statistical analysis of the Speed-groups, ANOVA revealed a significant effect of Group on the BP time window (*F*_1,43_ = 5.35, *p* < 0.05), which was larger in fast (−2.2 μV) than in slow (−1.3 μV) group; at the opposite, no differences emerged by analysis on the pN component (*F*_1,43_ = 0.33). For the Accuracy-groups, ANOVA on the BP revealed no significant effect of Group (*F*_1,39_ = 0.24), while the pN showed a main effect of Pool (*F*_1,39_ = 16.97, *p* = 0.0001) and a significant interaction Group × Pool (*F*_1,39_ = 5.26, *p* < 0.05). *Post hoc* revealed that the pN was larger (*p* < 0.05) in the inaccurate (−2.4 μV) than accurate (−1.4 μV) group. Moreover, the pN amplitude at the right side of the inaccurate group was larger than the left pN of both inaccurate (*p* < 0.0001) and accurate (*p* < 0.01) groups.

**Figure 3:**
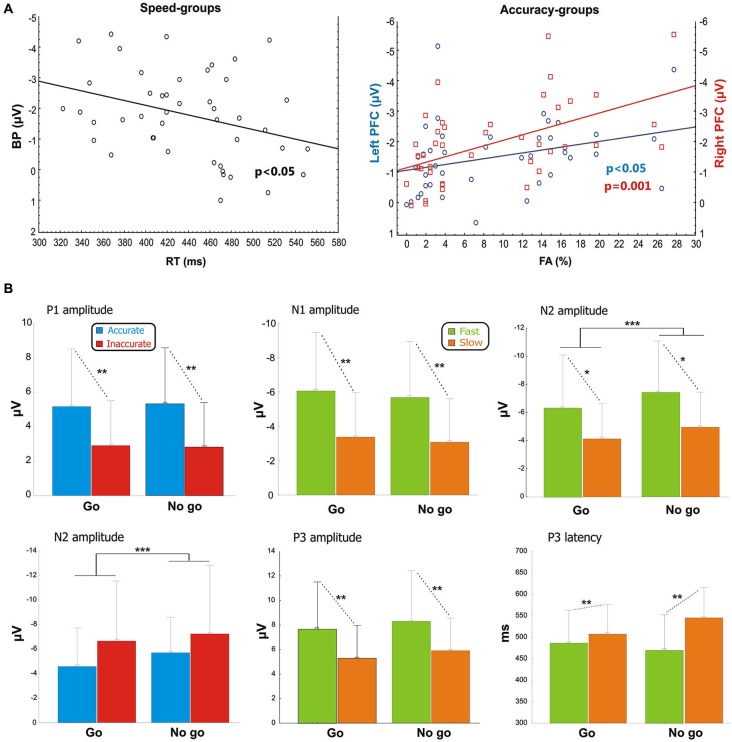
**(A)** Pre-stimulus activity. Left side: correlation scatterplot of the RT with the BP amplitude in the Speed-groups. Right side: correlation scatterplot of the FA with both the left and right PFC activity (indexed by the pN) in the Accuracy-groups. **(B)** Post-stimulus activity: means and standard deviations of the main ERPs components. From the upper left: P1 amplitude in Accuracy-groups; N1 amplitude in Speed-groups; N2 amplitude in Speed-groups; N2 amplitude in Accuracy-groups; P3 amplitude in Speed-groups; P3 latency in Speed-groups. * *p* < 0.05; ** *p* < 0.01; *** *p* < 0.001.

Pearson’s analysis showed that the BP amplitude of the Speed-groups correlated positively with the RTs (*r* = 0.33, *p* < 0.05); on the other hand, the analysis on the Accuracy-groups showed a significant correlation between the percentage of FAs and the pN activity of the left (*r* = −0.34, *p* < 0.05) and especially right (*r* = −0.48, *p* = 0.001) pools (Figure 3A). At the opposite, the correlations were neither significant between RTs and BP in the Accuracy-groups (*r* = 0.01, *p* > 0.05), nor between FAs and right pN in the Speed-groups (*r* = −0.08, *p* > 0.05). These results suggest that: (a) the larger the BP component, the faster the behavioral response; and (b) the larger the pN activity (especially on the right side), the worst the accuracy performance. Moreover, significant correlations emerged between the BP and the pN in both Speed- (*r* = 0.61, *p* < 0.0001) and Accuracy-groups (*r* = 0.4, *p* < 0.01), pointing to an interaction between SMA and right PFC activities.

### Post-stimulus ERPs

After stimulus onset, the P1 and N1 components peaked at about 110 and 170 ms, respectively, on bilateral parietal-occipital sites (PO7/PO8). At about 240 ms emerged the N2 peaking on medial frontal sites (Cz). Finally, the P3 component peaked between 470 and 545 ms over medial parietal and frontal sites. Statistical comparisons of the aforementioned components are shown in Figure 3B.

#### P1 component

For the Accuracy-groups, ANOVA showed a larger amplitude of the P1 in the accurate than inaccurate (*F*_1,39_ = 5.9, *p* = 0.01) group, and the Pearson’s test revealed a negative correlation between P1 amplitudes and FAs percentages (*r* = −0.35, *p* < 0.05) indicating that the amplitude was larger when the performance was more accurate.

#### N1 component

For the Speed-groups, the N1 component was larger in the fast than the slow group (*F*_1,43_ = 9.78, *p* < 0.01); further, its amplitude positively correlated with RTs in both Speed- (*r* = 0.48, *p* < 0.001) and Accuracy-groups (*r* = 0.36, *p* = 0.001), indicating that an enhancement of this component was associated with faster RTs in both cases.

#### N2 component

For the Speed-groups, ANOVA on the N2 component showed a main effect of Condition (*F*_1,43_ = 47.75, *p* < 0.0001), indicating that it was larger in no-go than go, and a main effect of Group (*F*_1,43_ = 5.03, *p* < 0.05), reflecting a larger N2 in the fast than slow group. For the Accuracy-groups, only the main effect of Condition (*F*_1,39_ = 19.98, *p* < 0.0001) was present, which was comparable to that observed for Speed-groups. Further, the N2 amplitude was positively correlated with the RTs in both Speed- (*r* = 0.38, *p* < 0.001) and Accuracy-groups (*r* = 0.47, *p* < 0.001), and negatively correlated with the FAs in both Speed- (*r* = −0.26, *p* < 0.001) and Accuracy-groups (*r* = −0.28, *p* < 0.001). In other words, larger N2 components were associated with faster RTs and more errors in both groups.

#### P3 component

ANOVA on the Speed-groups showed a main effect of Group for both P3 amplitude (*F*_1,43_ = 6.97, *p* = 0.01) and P3 latency (*F*_1,43_ = 7.46, *p* < 0.01), indicating an earlier and larger P3 component in the fast than the slow group. In the Accuracy-groups the effects on the P3 were not significant. Pearson’s analyses showed a negative sign correlation between RTs and P3 amplitude of both Speed- (*r* = −0.39, *p* < 0.001) and Accuracy-groups (*r* = −0.28, *p* < 0.01); further, the RTs was also positively correlated with the P3 latency of the Speed-groups (*r* = 0.27, *p* < 0.01) only. These data indicate that faster responses were associated with earlier and larger P3 peaks.

The results of the correlation analyses between electrophysiological data in the Speed- and Accuracy-groups are reported in Table 2. Overall, accuracy modulated the P1 and the N2 components in two opposite ways. The more accurate performance correlated with larger P1 amplitude and smaller N2 amplitudes. Speed modulated the N1, N2 and P3 components; the larger their amplitudes, the faster the RTs. For the P3 component, also the latency was related to RTs speed (the shorter P3 latency, the faster RTs).

**Table 2:** **Correlations (*r*-values) between ERP components in the Speed- and Accuracy-groups**.

	**Speed-groups**					**Accuracy-groups**		
	**pN**	**N1**	**dpP latency**	**P3**		**BP**	**P1**	**N2**
BP	0.61***	0.43**	0.33*	−0.57***	pN	0.4**	0.35*	0.43**

When not specified, the amplitude values are considered. *p < 0.05; **p < 0.01; ***p < 0.001.

### Differential waves

To enhance the go-related pP, the differential waves (go minus no-go) were calculated on the frontopolar derivations (Fp1, Fp2), limiting the analyses to the time window following the stimulus. By this method, the no-go condition acted as baseline for the go ERP in each subject: this procedure was motivated by the fact that the pP activity was closely associated to the response trials (i.e., Go); furthermore, the adopted spatial and temporal restrictions allowed us to isolate the known component without extending the observation to unknowable and interpretable waves.

**Figure 4:**
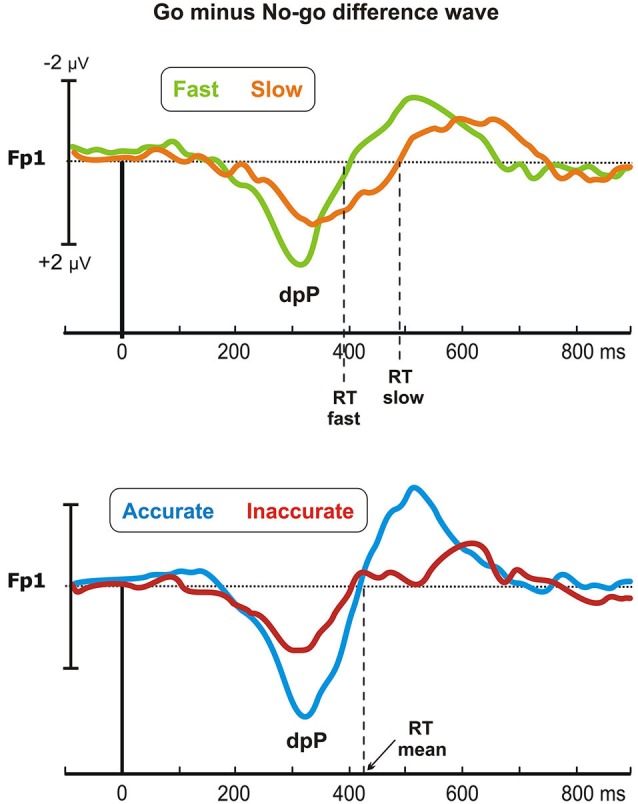
**Go minus no-go difference wave: the differential prefrontal positivity (dpP)**. Differential activity is reported for the left frontopolar electrode (Fp1) for both Speed- (top) and Accuracy-groups (bottom). Time 0 represents stimulus onset.

Figure 4 shows the difference waveforms (restricted to the post-stimulus period) over the left prefrontal site (Fp1), in which the dpP was largely pronounced. In the Speed-groups, the dpP of the fast group started approximately 60 ms earlier than the slow group, and this difference partially remains until the peak, which was reached at 309 and 351 ms by the fast and slow group, respectively. Furthermore, the peak was larger in the fast than slow group. On the other hand, the accurate group had larger dpP than the inaccurate group, but latency differences were not present. These trends were confirmed by the ANOVAs, which in the Speed-groups revealed significant effects on the onset latency (*F*_1,43_ = 20, *p* < 0.0001), peak latency (*F*_1,43_ = 8.3, *p* < 0.01) and peak amplitude (*F*_1,43_ = 7.8, *p* < 0.01). For the Accuracy-groups, only the peak amplitude was different between groups (*F*_1,39_ = 5.5, *p* < 0.05).

Pearson’s analyses showed that the RTs were positively correlated with the onset latency of the Speed-groups (*r* = 0.46, *p* = 0.001), and with the peak latency of both Speed- (*r* = 0.39, *p* < 0.01) and Accuracy-groups (*r* = 0.37, *p* < 0.05). Moreover, significant negative correlation emerged between the dpP amplitude and the RTs of the Speed-groups (*r* = −0.4, *p* < 0.01), confirming that the larger the dpP, the faster the response. Overall, this differential wave enhancing go-related processing at prefrontal level was a sensitive marker of the efficiency of the decision processing in both Speed- and Accuracy-groups.

## Discussion

This study aimed at identifying the neural processing stages associated with the SAT using a novel approach, *i.e*., selecting subjects based on their spontaneous speed or accuracy tendency rather than manipulating speed or accuracy requirements. Moreover, we recorded the frontal activity with a much more dense electrode array than previous electrophysiological studies (Osman et al., 2000; van der Lubbe et al., 2001; Sangals et al., 2002; Band et al., 2003; Rinkenauer et al., 2004) allowing discrimination of two different frontal activities in the temporal window before stimulus onset. Finally, we considered the characteristics of ERP components after stimulus, highlighting different levels of perceptual processing associated with response speed or response accuracy.

### Pre-stimulus activities

The anticipatory brain activities (the BP and pN components) showed group differences depending on the speed or the accuracy of the subsequent motor response. Fast and slow groups (matched in accuracy) had different BP amplitudes and similar pN amplitudes; at the opposite, accurate and inaccurate groups (matched in speed) had different pN amplitudes and similar BP amplitudes. The sources of these components were located in different areas of the frontal cortex: the SMA for the BP component (Di Russo et al., 2005; Berchicci et al., 2012), and the PFC for the pN (Berchicci et al., 2013; Di Russo et al., 2013a,b). An enhanced SMA activity in the last half second before the stimulus onset characterized subjects with fast responses with respect to slow subjects. By contrast, an enhanced rPFC activity starting 250 ms before the stimulus onset characterized inaccurate subjects with respect to very accurate subjects. Correlations between SMA amplitude and RTs on one side, and between rPFC amplitude and accuracy on the other further support the different roles played by these two frontal areas into speed and accuracy processing. However, it is noteworthy that the pre-stimulus activities were correlated (the larger the BP, the larger the pN) within both Speed and Accuracy groups, pointing to a stable relationship between SMA and rPFC activity.

The enhanced SMA activity was associated with speed instructions in fMRI (Forstmann et al., 2008b; Ivanoff et al., 2008; van Veen et al., 2008), MEG (Wenzlaff et al., 2011) and EEG (Brunia and Vingerhoets, 1980; Band et al., 2003; Rinkenauer et al., 2004) studies. Neurophysiologically, larger SMA activity under speed constrain might contribute to overcome the tonic inhibition provided by the output nuclei of basal ganglia (Lo and Wang, 2006). Present findings showed that the subjects with a spontaneous tendency to be fast had an enhanced SMA activity starting 500 ms before the stimulus onset, suggesting that baseline activity increased in fast performers. Indeed, a reduced baseline-to-threshold distance could account for the shorter time needed to reach a motor response (Bogacz et al., 2010).

On the other hand, it is still a matter of debate the role played by prefrontal areas in the SAT processing, although the engagement of the rPFC in the response accuracy is supported by studies on the response inhibition (Garavan et al., 1999, 2002; Stuss et al., 2002), and the ability to differentiate correct stimuli (Stuss et al., 2003), especially in tasks requiring sustained attention (Wilkins et al., 1987; Glosser and Goodglass, 1990) such as the present one. Present findings indicate that the rPFC activity starting 250 ms before the stimulus onset was accuracy-related (larger in the inaccurate than accurate group).

Based on present results, we propose that: (1) speed and accuracy tendencies are settled by the activity of two distinct frontal areas (the SMA and rPFC, respectively) long before the stimulus onset (for this reason called “baseline”); and (2) although there is a trade-off between SMA and rPFC activities (i.e., the BP and pN were correlated), it is not total: indeed, each group was marked by amplitude differences in only one component, without affecting the other. Thus, two interacting but separate neurocognitive systems may represent the basis of the individual tendencies underlying the *baseline* modulation of different baseline-to-threshold systems. In the “speed system” (modulated by the SMA), the increased baseline could lead to fast responses, while in the “accuracy system” (modulated by the rPFC) the increased baseline could lead to inaccurate performance, because of the reduced possibility of accumulating sufficient evidences until threshold reaching. Thus, we propose that SAT is the result of the co-activation of the two interacting systems. Indeed, considering the anatomo-functional connections between the SMA and rPFC (for a review see Aron, 2011), it could be proposed that an increased baseline activity in the SMA-rPFC network leads to fast and inaccurate performance, while the decreased baseline accounts for the trade-off in the sense of slow and accurate responses.

### Post-stimulus activities

Data on post-stimulus activities are consistent with the view that accuracy- or speed-related individual tendency might affect also the activity of visual cortical areas. We observed a dissociation of the two visual components P1 and N1, which had larger amplitudes in the accurate and fast groups than slow and inaccurate groups, respectively. The dissociation was further confirmed by the correlation analyses, showing that larger P1 amplitude was associated with high accuracy, and larger N1 amplitude was associated with high speed.

A vast literature showed that spatial attention produces an amplification of stimulus-evoked activity in extrastriate areas and posterior parietal cortex (PPC) during the 80–250 ms following the stimulus onset (Luck et al., 1990; Clark and Hillyard, 1996; Wijers et al., 1997; Hillyard et al., 1998; Martínez et al., 1999; Di Russo et al., 2003). These studies support the “early selection” theories of visual-spatial attention (Bashinski and Bacharach, 1980; Johnston and Dark, 1986; Downing, 1988; Hillyard and Anllo-Vento, 1998); however a different role of the P1 and N1 components should be considered (Luck et al., 1990). The P1 component enhancement represents facilitation at the early sensory processing level for items presented at attended location (Di Russo et al., 2003), while the N1 component is associated with the discrimination processes within the focus of attention (Luck et al., 1990; Vogel and Luck, 2000). In addition to the modulation of the extrastriate areas, visual attention control relies on a network of cortical and subcortical regions, including the DLPFC and PPC, the anterior cingulate gyrus, and the pulvinar nucleus of the thalamus (Mesulam, 1990; Nobre et al., 1997). Thus, it is likely that the modulations of the visual areas observed in the present study are part of a perceptual decision-making process, starting with pre-stimulus baseline adjustments and ending up with the response threshold reaching. We propose that individual speed- and accuracy-oriented neural strategies provide “bias signals” that exert a selective amplification of sensory information flow in different visual pathways. Support to this hypothesis comes from a single cell recording (Heitz and Schall, 2012) showing that the SAT-related cues induced a shift of baseline firing rates in the visually responsive neurons of the frontal eye field (FEF). At the same time, under the framework of the drift-diffusion models, recent studies (Rae et al., 2014; Zhang and Rowe, 2014) suggest that not only the boundary threshold but also other parameters are affected by the speed or accuracy; for example, it was proposed that emphasis on accuracy increased the allocation of attention on the task (i.e., the drift rate) and the non-decision time, i.e., the time reserved to the stimulus encoding. These latter hypotheses are consistent with the present findings, pointing to a greater allocation of visual-spatial attention in the accurate group, as revealed by the P1 amplitude. Further studies are needed to shed light into the brain networks underlying the speed- and accuracy-oriented perceptual processes, as indexed by the P1-N1 modulation.

Difference between groups was also observed for the N2 component: it was larger in the fast than slow group, showing also the “typical” no-go enhancement (e.g., Donkers and van Boxtel, 2004). The N2 modulation is generally described as an index of inhibitory control (e.g., van Boxtel et al., 2001) or as conflict monitoring between go and no-go stimuli (Nieuwenhuis et al., 2003; Donkers and van Boxtel, 2004). However, we will not discuss the N2 data in these terms, because in a recent study (Di Russo et al., 2013b) combining ERP and fMRI measures using the same paradigm of the present one, we found that the no-go condition did not produce larger activity than the go condition in any brain areas, indicating that the no-go N2 cannot be the expression of extra (inhibitory or conflict-related) activity, but more likely the summation of negative and positive waveforms originating in premotor, prefrontal and parietal areas in the same time period (200–400 ms after the stimulus). Further studies are required to clarify this issue, which is outside the scope of present work.

The P3 component, usually described as an index of the stimulus categorization process (Mecklinger and Ullsperger, 1993), started earlier and was larger in the fast than slow group, whereas no differences emerged between accurate and inaccurate groups. The correlation analyses further confirmed the relationship between the RT and the P3 component, suggesting that the P3 could also provide an estimation of the stimulus evaluation time that is closely related to the response processing time.

Finally, are crucial the effects found on the prefrontal pP. We confirmed that this newly discovered components, compared to no-go, is larger in the go condition as previously described by our group (Di Russo et al., 2013a,b; Berchicci et al., 2014). The neural generator of the pP was localized in the anterior Insula in a study combining fMRI and ERP data collected with the same task used in the present study (Di Russo et al., 2013b), and its function would be to trigger the response when enough information are accumulated. Other studies showed that insular activation indicates the stimulus-response (S-R) association to guide response selection (Boettiger and Dand’Esposito, 2005), and reflects both self and motor awareness (Berti et al., 2005). In the present study, we additionally adopted the subtraction method to better focus on the pP modulation on prefrontal sites: the main risk of this procedure is to compare different activities acting in the same period. For this reason, our analyses and interpretation were limited to the differential activity resulting from the frontopolar derivations in the time window following the stimulus. In line with our predictions, we observed a positive component, called dpP, peaking at about 330 ms after the stimulus: thus, differential analyses further confirmed the presence of a positive activity closely related to the response execution, as previously observed in other studies (Di Russo et al., 2013a,b; Berchicci et al., 2014). Taking into account these views and the present data, we suggest that the dpP might reflect the S-R mapping finalized to the response execution in a perceptual discrimination task, representing the final stage of the decision process before the movement onset. Analyses on the dpP showed that the latency of this differential wave reflects the speed of the decision-making processing. Indeed, the dpP started earlier in the fast than slow group (see Figure 4), explaining about the 60% of the RT difference between the two groups. Moreover, the dpP wave was larger in both fast and accurate groups than their respective counterparts, suggesting that its amplitude reflects the efficiency of the decision process in both cases.

### Speed and accuracy decision systems: an integrative view

In summary, present results showed different brain activities both before and after stimulus onset in Speed- and Accuracy-groups. Pre-stimulus activity in the SMA and rPFC seems to reflect the baseline modulation of the speed and accuracy decision systems: they are interacting, as revealed by present analyses and anatomo-functional connections between SMA and rPFC (for a review see Aron, 2011). Thus, we suggest that the typical trade-off between response speed and accuracy is accounted by the baseline activity in the SMA-rPFC network. A baseline increase in this network could prepare subjects to fast and inaccurate performance, while a reduced baseline may predict slow and accurate performance because of the greater baseline-to-threshold distance in both the speed and accuracy systems. In addition, we showed that the speed and accuracy baselines can also be separately modulated, leading to either high or low group performance in one system without affecting (or affecting very little) the other, as indicated by comparable mean performance in the other system. Thus, as previously suggested, the two systems should be considered interacting but not totally dependent. Finally, after stimulus onset, ERP components reflecting perceptual processing, S-R mapping and stimulus categorization were also differentially affected by speed and accuracy idiosyncratic tendencies.

**Figure 5:**
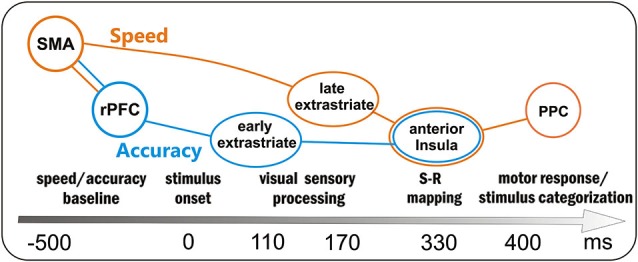
**Sketch of the processing in the preparation-perception- action cycle and associated brain areas as a function of time (not scaled)**. Obviously the same brain areas were involved in both speed- (orange) and accuracy (blue)- processing; however, the activity of some areas was more affected by either one or the other condition: the orange and blue lines depict the two main flows within speed and accuracy systems. SMA = supplementary motor area, rPFC = right prefrontal cortex, PPC = posterior parietal cortex.

Overall, the present study suggests that the motor response in a perceptual discrimination task should be considered as the final output of a series of neurocognitive processes starting long before the stimulus onset. For this reason, and based on our results, we sketched in Figure 5 the time course of the main processes supporting the go/no-go task. Obviously, all brain areas were active in both speed and accuracy processing; however, some areas were more involved in the speed with respect to accuracy system, and we tried to distinguish them by using different colors. Before stimulus onset the baseline activity of the speed and accuracy systems was modulated by the SMA (reflected by the BP) and the rPFC (reflected by the pN), respectively. Even if the activity of these prefrontal areas was correlated (accounting for the interaction between the two systems), the larger SMA activity marked only the fast group, while the larger rPFC activity marked only the inaccurate group. About 110 ms after the stimulus onset, the early sensory processing of the extrastriate areas (P1 component) was modulated by the accuracy level, with the accurate group focusing greater attention to the attended location. Immediately after, extrastriate visual and parietal areas (N1 component) showed a more intense processing, likely corresponding to the discrimination stage, in the fast than the slow group. Because of this enhanced sensory processing, the response-oriented S-R mapping in the anterior Insula (as reflected by the dpP) was reached earlier and Insula activity was larger in fast with respect to slow group; moreover, also accuracy affected the anterior Insula activity (larger dpP in the accurate than inaccurate group), although its activity was not directly correlated with the rPFC modulation. In a time window around 400 ms, the activity corresponding to the stimulus categorization in the PPC (P3 component) and response execution in the case of go stimuli, was especially affected by response speed.

Summarizing, present data suggest that the behavioral speed-accuracy trade-off (SAT) is explained by the neurocognitive processing of two “decision systems”, starting to work before the stimulus appearance and reflecting the neural substrate of idiosyncratic tendencies. A limitation of the present study is that we did not observe if speed is traded for accuracy (or vice versa) at a single-subject level, and we matched the groups by *a posteriori* criteria based on behavioral performance; however, considering that task instructions equally emphasized speed and accuracy, we thought it might represent a sort of spontaneous sorting, enhancing idiosyncratic individual tendency.

## Conflict of interest statement

The authors declare that the research was conducted in the absence of any commercial or financial relationships that could be construed as a potential conflict of interest.
